# The genomic diversity and antimicrobial resistance of Non-typhoidal *Salmonella* in humans and food animals in Northern India

**DOI:** 10.1016/j.onehlt.2024.100892

**Published:** 2024-09-12

**Authors:** Jaspreet Mahindroo, Duy Pham Thanh, Harpreet Kaur, To Nguyen Thi Nguyen, Trang Hoang Thu Nguyen, Megan E. Carey, Ritu Verma, Balvinder Mohan, Siddhartha Thakur, Stephen Baker, Neelam Taneja

**Affiliations:** a Department of Medical Microbiology, Post Graduate Institute of Medical Education and Research, Chandigarh, India; b MRC Centre for Global Infectious Disease Analysis, School of Public Health, Imperial College London, London, UK; c Oxford University Clinical Research Unit, Wellcome Trust Major Overseas Programme, Ho Chi Minh City, Viet Nam; d Department of Infectious Diseases, Central Clinical School, Monash University, Melbourne, VIC 3004, Australia; e Department of Infection Biology, London School of Hygiene & Tropical Medicine, London, UK; f College of Veterinary Medicine, North Carolina State University, Raleigh, NC, USA; g Cambridge Institute of Therapeutic Immunology and Infectious Disease (CITIID), Department of Medicine, University of Cambridge, Cambridge, United Kingdom; h IAVI, Chelsea & Westminster Hospital, London, UK

**Keywords:** Non-typhoidal *Salmonella*, Whole genome sequencing, MLST, Human-animal interface, Antimicrobial resistance, India

## Abstract

**Introduction:**

Non-typhoidal *Salmonella* (NTS) serovars are the leading global cause of gastroenteritis and have established reservoirs in food animals.

**Gap statement:**

Due to a lack of surveillance, there is limited information on the distribution of NTS serovars in India.

**Aim:**

Here, we investigated the epidemiology, sequence types, serovar distribution, phylogenetic relatedness, and antimicrobial resistance patterns of NTS in humans and animals across a large geographic area in Northern India.

**Methodology:**

We collected stool samples from patients with diarrhea who presented to 14 laboratories in Chandigarh and from five states in India (Punjab, Haryana, Uttarakhand, Himachal Pradesh, and Rajasthan). We sequenced the genomes and analyzed 117 NTS organisms isolated from humans and animals. Minimum inhibitory concentrations (MICs) were estimated using a Vitek2 system.

**Results:**

The prevalence of NTS in participants presenting to our study with diarrhea was 1.28 %, affecting all age groups. All NTS caused moderate to severe diarrhea. We found a high diversity of serovars with considerable serovar and sequence types (STs) overlap and phylogenetic closeness between isolates from human infections and food animals. We report serovars such as *S.* Agona, *S.* Bareilly, *S.* Kentucky, *S.* Saintpaul, and *S.* Virchow, causing human infections from north India for the first time. Among the different food-producing animals, pigs appeared to be a key source of human infections. Twenty-eight percent (28 %) of the NTS isolates were multi-drug resistant (MDR), and human isolates showed a higher proportion of resistance. A higher level of contamination of meat samples in our study (8.4 %) potentially suggests a close association of NTS serovars with the food chain and high transmission risk in north India.

**Conclusions:**

This study provides information on AMR genes and plasmid replicons associated with different serovars and highlights the role of food animals in AMR dissemination in our region.

## 1 Introduction

Gastroenteritis is the primary clinical manifestation of human infection with non-typhoidal *Salmonella* (NTS) organisms [1]. NTS is estimated to cause 94 million cases of gastroenteritis and 1,150,000 deaths each year globally, making them among the leading global causes of diarrhea [2]. More than 2500 NTS serovars have been described as originating from a broad range of hosts and commonly colonizing the gastrointestinal tract of animals farmed for food [3]. Some NTS serovars are host-specific, residing in only one or very few animal species. Still, the majority are promiscuous and can cause cross-species transmission and trigger human disease. According to estimates from the World Health Organization (WHO) Salm-Survsurveillance network, *Salmonella Enteritidis* (*S. enteritidis*) was the most common cause of *Salmonella*-associated diarrhea worldwide in 2001–2002, followed by *Salmonella Typhimurium* (*S. typhimurium*) and *Salmonella* Newport (*S.* Newport) [4]. In a recent meta-analysis of *Salmonella* serovars isolated from food animals, *S. typhimurium* was the most prevalent serovar in all food matrices [5].

There are limited data on the contemporary distribution of NTS serovars across India. This paucity of information is due to a lack of surveillance of food-borne illnesses, and serotyping of NTS isolates is seldom performed when organisms are isolated. Historically, numerous serovars in zoonotic reservoirs have been reported in India. The National *Salmonella* and *Escherichia* Centre (NSEC) reported *Salmonella* Richmond (*S.* Richmond), *S. typhimurium*, and *Salmonella* Weltevreden *(S.* Weltevreden) to be the most commonly isolated NTS serovars from poultry in 1965, with *Salmonella* Poona (*S.* Poona) being the most frequently isolated from pigs [6]. More recently, in 2005, *S. typhimurium* remained common in poultry along with *S. enteritidis* and *Salmonella* Gallinarum (*S. gallinarum*); *Salmonella* Dublin *(S.* Dublin*)* was the predominant serovar in other animals [7]. *Salmonella* Kentucky (*S.* Kentucky) and *S. typhimurium* are currently considered to be the most predominant serovars found in animals in India [8].

As is the current trajectory for many Gram-negative bacteria, there has been a substantial increase in antimicrobial resistance (AMR) in NTS organisms. While this increase in AMR is variable between serovars,multi-drug resistance (MDR; resistance to chloramphenicol, ampicillin, and co-trimoxazole) has spread globally since the 1980s and 1990s. The WHO identified fluoroquinolone-resistant *Salmonella* as a high-priority pathogen, requiring the development of new effective antimicrobials [9] [10]. Again, antimicrobial susceptibility data from NTS from India are limited and rarely obtained from the veterinary sector. Available data suggest that aminoglycoside resistance is common, ranging from 15 % to 58 %, depending on the source of isolation [11]. Resistance to fluoroquinolones and cephalosporins is uncommon, but a recent study has found the prevalence of ciprofloxacin resistance in NTS to be >98 % [11]. *S. typhimurium* and *S*. Kentucky, resistant to many critical antimicrobials, have been isolated from poultry [8,12]. In 2004, we reported 18.5 % resistance to ciprofloxacin in the NTS isolates [13,14]. The prevalence of ciprofloxacin resistance increased along with the prevalence of resistance to third-generation cephalosporins in the next decade [14].

Here, aiming to investigate the NTS at the human-animal interface in Northern India, we consecutively collected samples from humans and animals in five northern Indian states and isolated NTS. We used whole-genome sequencing (WGS) to determine the distribution of serovars, sequence types (STs), AMR gene composition, and plasmid profiles and to assess phylogenetic relatedness.

## 2 Materials and methods

### 2.1 Sample collection

#### 2.1.1 Human samples

**Fig. 1:**
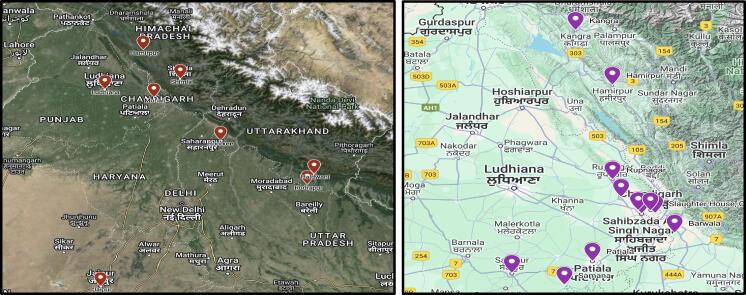
Human and animal samples collection sites in this study (produced from Google Maps). (A) showing the collection sites for human samples and (B) animal collection sites.

A sustained surveillance was carried out from March 2015 to February 2018 for human diarrhoeal disease at the Post Graduate Institute of Medical Education and Research (PGIMER Chandigarh) and network laboratories in the states of Punjab, Haryana, Rajasthan, Uttarakhand and Himachal Pradesh. 14 labs across North India participated in the study (Fig. 1). Cases referred to PGIMER were also included (n-56). PGIMER is one of the largest tertiary care hospitals in North India and serves patients from across Punjab, Jammu Kashmir, Himachal Pradesh and Haryana. Diarrhoea was defined as the passage of three or more liquid/semi-liquid stools. All hospitalacquired diarrhoea cases (diarrhoea occurring in cases after 48h of presentation to the health care facility) were excluded. Stool samples from patients were collected in a sterile container and transported to PGIMER Chandigarh in Cary-Blair in a cold chain. The samples were processed immediately upon receipt. Before sample collection, informed consent from all patients or their guardians for children, along with detailed clinical history, information about food consumption, and source of water supply, was collected by laboratory technicians under the supervision of microbiologists at the time of sample collection. Vesikari severity score was used to assess the severity of diarrhoea. It was calculated based on the number of diarrheal and vomiting episodes, duration of illness, fever and dehydration status [15, 16].

#### 2.1.2 Animal samples

Concurrently, we conducted cross-sectional sampling of food animals, meat products and farm animals (sheep, goats, pigs and chickens) in the states of Punjab, Haryana, Himachal Pradesh and Chandigarh from markets and farms in the same areas from where human samples were collected (Fig. 1). The meat shop and farm owners were approached. Those who agreed to provide samples were included in the study. A total of 27 poultry farms [big with a capacity of 10000-15000 birds (*n* = 12) and moderate-sized with a capacity of housing 2000-3000 birds (15)], eight pig farms, and 27 sheep/goat farms provided permission for sample collection. A total of 112 abattoir facilities were approached, of which 75 chicken, 16 pig, and 50 goat/sheep shops allowed animal meat sample collection [17]. In a repeated cross-sectional manner, samples from goats and pigs were also collected from the slaughterhouse in Chandigarh. We collected samples every Wednesday for 30 weeks, from March 2014 to October 2014. Slaughterhouse under the Municipal Corporation of Chandigarh is a mechanical abattoir that caters to Chandigarh and the neighbouring cities of Mohali, Kharar, Panchkula, Manimajra, Zirakpur, Balongi, Meat market sector 21 Chandigarh. Up to 250 goats/sheep and 25-30 pigs are slaughtered daily. A food inspector visually checks the meat that is then transported to shops in Chandigarh, Punjab, and Haryana in a controlled temperature transport system.

The samples were collected in sterile containers and transported in a cold chain to PGIMER, where they were processed immediately. The total number of samples collected was 906, of which 487 were animal stools/intestinal contents and 352 were meat samples.

#### 2.1.3 Salmonella isolation

All samples were collected in Cary Blair in sterile containers, transported to PGIMER in temperature-controlled conditions, and processed immediately. A loopful (∼10 μl) human stool sample was inoculated onto MacConkey and XLT4 agar and selectively enriched in Rappaport-Vassiliadis broth for NTS isolation from human faecal samples. For NTS isolation from animal samples, ∼25 g of meat and ∼ 10 g of stool samples were inoculated into buffered peptone water and selectively enriched in Rappaport-Vassiliadis broth and sub-cultured onto MacConkey and XLT4 agar. Non-lactose fermenting colonies were confirmed as *Salmonella* spp. by use of the MALDI-TOF bacterial identification system (Bruker Daltonics, Germany).

#### 2.1.4 Whole genome sequencing

For WGS, fresh overnight growth on Nutrient agar (Oxoid, Hampshire, England, U·K) was used for DNA extraction by Wizard genomic DNA purification kit (Promega Corporation, Madison, USA) according to the manufacturer's protocol. The quality of DNA was assessed by a Nanodrop 2000 (ThermoFisher Scientific Wilmington, DE, USA) and quantified using a Quantas fluorometer (Promega Corporation, Madison, USA). Genomic libraries were prepared using Illumina's Nextera XT DNA Library Prep kit (Illumina, San Diego, California, USA). The libraries were sequenced on the Illumina MiSeq platform with a V3–300 reagent cartridge (Illumina, San Diego, California, USA) to generate 150 bp paired-end reads.

#### 2.1.5 Sequence analysis

The read quality of the genome sequences was assessed by FASTQC [18]. The serovars were identified from the raw reads using Seq-Sero software version 2.0 [19]. *In-silico* MLST (Achtman 7 gene MLST Scheme), plasmid replicons by plasmid finder database, and AMR determinants were identified by the ARG-Annot database of SRST2 [20]. To determine the phylogenetic relatedness between the isolates, raw reads were mapped to the reference genome of *S. enterica* Kentucky ST198 strain PU131, accession number CP026327, using the RedDog pipeline V1beta.10.3 (https://GitHub.com/katholt/RedDog). The pipeline mapped the raw reads using Bowtie2 version 2.2.3 [21], and SNPs were called from the alignments generated using SAMtools version 1.1.19 [22]. A maximum likelihood phylogenetic tree was generated using five independent runs of Randomized Axelerated Maximum Likelihood (RAxML) [23] with a general time-reversible (GTR) model of substitution [24] and 100 bootstrap pseudo-replicates. Substitutions in the quinolone resistance determining region (QRDR) codons in the *gyrA* and *parC* genes were identified from the allelic variant output file from the RedDog pipeline. The resultant tree was visualized using the Interactive Tree of Life (iTOL) [25].

#### 2.1.6 Antimicrobial susceptibility testing

Minimum inhibitory concentrations (MICs) of different antibiotics for *Salmonella* were estimated using a Vitek2 system using an N240 card (bioMérieux, San Antonio, Texas, USA) Table 1. MIC values for colistin and ciprofloxacin were estimated using *E*-strip method (*bioMérieux*, Lyon, France) on Mueller Hinton agar (Difco, Sparks, MD, USA). MDR organisms were defined as resistant to at least one antimicrobial in three or more classes of antimicrobials [26].

**Table 1:** List of antibiotics used in MIC testing.

**Sr No.**	**Antibiotics**
1	Amikacin (2–64 μg/ml)
2	Aztreonam (1–64 μg/ml)
3	Cefepime (1–64 μg/ml)
4	Ceftazidime (1–64 μg/ml)
5	Ciprofloxacin (0.25–4 μg/ml)
6	Colistin (0.5–16 μg/ml)
7	Gentamicin (1–16 μg/ml)
8	Imipenem (0.25–16 μg/ml)
9	Levofloxacin (0.12–8 μg/ml)
10	Meropenem (0.25–16 μg/ml)
11	Piperacillin (4–128 μg/ml),
12	Piperacillin-tazobactum (4/4–128/4 μg/ml)
13	Rifampicin (2–32 μg/ml)
14	Ticarcillin (8–128 μg/ml),
15	Ticarcillin-clavulanic acid (8/2–128/2 μg/ml)
16	Tobramycin (1–16 μg/ml)
17	Trimethoprim-sulfamethoxazole (20[1/19]-320[16/302] μg/ml)

### 2.2 Statistical analysis

Fisher's exact test was used to compare the significant difference in resistance levels among human and animal isolates. Statistical analysis was performed with GraphPad Prism version 8.4.3 for Windows (GraphPad Software, San Diego, California, USA), and *p* values less than 0.05 were considered significant.

## 3 Results

### 3.1 Baseline data

We isolated 117 NTS organisms comprised of 25 organisms isolated from patients with diarrhea (from 1968 faecal samples, 1.3 % positive) and 87 animal samples (from 906 samples, 9.6 % positive) (Table 2, Table 5). In addition to the 25 NTS human isolates, we cultured a small number of typhoidal *Salmonella* organisms: *Salmonella Paratyphi* A (*S.*Paratyphi A, *n* = 2) and *Salmonella Typhi* (*S. typhi*,*n* = 4). The human isolates were obtained from Chandigarh, Punjab, Haryana, Himachal Pradesh, and Uttarakhand. Most Salmonella cases were detected in adolescents and adults (17/25; 68 %); 23 was the median patient age (Table 2). We isolated 32 % (8/25) from children under twelve years and 16 % (4/25) from adults 60 years and older. Using the Vesikari scale, 40 % (10/25) of patients had severe diarrhea (score of 10 or higher), while 60 % (15/25) had moderately severe diarrhea (score between 7 and 10). Fever with acute gastroenteritis (*n* = 16/25; 64 %) was the most common presentation; 88 %, 80 %, and 64 % had abdominal pain, vomiting, and dehydration, respectively. A definite history of food-borne illness could be confirmed in 48 % (12/25) cases (Table S1). Ciprofloxacin/ceftriaxone was used to treat moderate to severe cases.

**Table 2:** Age and gender-wise distribution of patients from different regions.

**Region**	**Age group (years)**	**Male (%)**	**Female (%)**	**Total (%)**
Chandigarh	0–2	96 (14.81)	49 (7.56)	145 (22.37)
	>2–5	36 (5.55)	17 (2.62)	53 (8.17)
	>5–15	47 (7.25)	31 (4.78)	78 (12.03)
	>15–40	140 (21.6)	110 (16.9)	250 (38.58)
	>40	60 (9.25)	62 (9.56)	122 (18.82)
		379 (58.48)	269 (41.5)	648
	Details not available			0⁎
*n* = 648	Sub-total			**648**
Punjab	0–2	101 (26.16)	39 (10.1)	140 (36.26)
	>2–5	31 (8.03)	10 (2.5)	41 (10.6)
	>5–15	33 (8.54)	25 (6.47)	58 (15.02)
	>15–40	66 (17.09)	31 (8.03)	97 (25.12)
	>40	32 (8.29)	18 (4.6)	50 (12.9)
		263 (68.13)	123 (31.8)	386
	Details not available			0⁎
*n* = 386	Sub-total			**386**
Haryana	0–2	77 (22.64)	49 (14.4)	126 (37.05)
	>2–5	21 (6.17)	12 (3.52)	33 (9.7)
	>5–15	17 (5)	10 (2.94)	27 (7.9)
	>15–40	42 (12.35)	45 (13.2)	87 (25.58)
	>40	36 (10.58)	31 (9.11)	67 (19.7)
		193 (56.76)	147 (43.2)	340
	Details not available			0⁎
*n* = 340	Sub-total			**340**
Rajasthan	0–2	10 (11.49)	3 (3.44)	13 (14.9)
	>2–5	2 (2.29)	2 (2.2)	4 (4.59)
	>5–15	4 (4.59)	7 (8.04)	11 (12.6)
	>15–40	18 (20.68)	21 (24.13)	39 (44.8)
	>40	13 (14.94)	7 (8.04)	20 (22.9)
		47 (54.02)	40 (45.9)	87
	Details not available			0⁎
*n* = 87	Sub-total			**87**
Himachal Pradesh	0–2	55 (21.4)	34 (13.2)	89 (34.6)
	>2–5	14 (5.4)	8 (3.1)	22 (8.5)
	>5–15	9 (3.5)	9 (3.5)	18 (7)
	>15–40	37 (14.39)	32 (12.45)	69 (26.8)
	>40	34 (13.2)	25 (9.7)	59 (22.9)
		149 (57.9)	108 (42.02)	**257**
	Details not available			5⁎
*n* = 262	Sub-total			**262**
**Area**	**Age group**	**Male**	**Female**	**Total**
Uttarakhand	0–2	12 (6.62)	12 (6.6)	24 (13.2)
	>2–5	11 (6.07)	8 (4.4)	19 (10.5)
	>5–15	31 (17.1)	12 (6.62)	43 (23.75)
	>15–40	25 (13.8)	26 (14.36)	51 (28.17)
	>40	25 (13.8)	19 (10.49)	44 (24.3)
		104 (57.4)	77 (42.5)	**181**
	Details not available			8⁎
*n* = 189	Sub-total			**189**
Others⁎	0–2	10 (17.8)	0	10 (17.8)
	>2–5	1 (1.8)	3 (5.4)	4 (7.2)
	>5–15	2 (3.6)	2 (3.6)	4 (7.2)
	>15–40	20(35.7)	9 (16.0)	29 (51.7)
	>40	7 (12.5)	2 (3.6)	9(16.1)
	Total	40 (71.4)	16 (28.6)	**56**
	Details not available			0⁎
Grand total				1968

⁎ referred cases.

The majority of NTS from animal samples were isolated from pigs (*n* = 50/87; 57.5 %), followed by chickens (*n* = 28/87; 32.2 %) and goats (*n* = 9/87; 10.3 %). The animal isolates were obtained from stool (*n* = 57/487; 11.7 %) and meat samples (35/419;*n* = 8.4 %).

### 3.2 Serovar and sequence type distribution

All isolates were subjected to WGS, and we used these data to infer serovars and STs. 18 NTS serovars comprising 22 STs were detected (Table 3). The most common human serovars were *S.* Kentucky (6/25, ST198), followed by *Salmonella* Saintpaul (*S.*Saintpaul) (*n* = 3/25, ST49 & ST27), *S. typhimurium* (n = 3/25, ST36), *S. enteritidis* (n = 2/25, ST11), and *Salmonella* Virchow (*S.* Virchow) (n = 2/25, ST16 and ST197). Singletons included *Salmonella* Agona (*S.* Agona) (ST13), *Salmonella* Bareilly (*S.* Bareilly) (ST203), *Salmonella* Infantis (*S. infantis*) (ST32) and *S.* Newport (ST166).

**Table 3:** Serotype distribution for *Salmonella* serovars.⁎

**Serotype (n)**	**Human**	**Goat**	**Pig**	**Chicken**	**Sequence type (MLST)**
Agona (22)	1	2	18	–	13
	–	–	1	–	2332
Anatum (15)	–	2	13	–	64
Bareilly (1)	1	–	–	–	203
Braenderup (6)	–	–	6	–	22
Enteritidis (2)	2	–	–	–	11
Indiana (1)	–	–	1	–	2040
Infantis (2)	1	–	–	1	32
Kentucky (30)	8	2	–	18	198
	–	1	1	–	314
Newport (4)	1	–	–	–	166
	–	1	2	–	31
Paratyphi A (2)	1	–	–	–	129
	1	–	–	–	85
Reading (1)	–	–	1	–	93
Rissen (1)	–	–	1	–	469
Typhi (4)	2	–	–	–	2
	1	–	–	–	1
Typhimurium (10)	–	–	–	6	19
	3	–	1	–	36
Saintpaul (4)	2	–	–	–	49
	1	–	1	–	27
Stanley (1)	–	–	1	–	2458
Virchow (2)	1	–	–	–	16
	1	–	–	–	197
Weltevreden (4)	–	1	1	2	365
Unknown (5)	3	–	2	1	
Total (117)	30	9	50	28	

⁎ Unknown: Complete formulae were not predicted.

Among the organisms isolated from the food animals, *S.* Kentucky was the predominant serotype (*n* = 22/87; ST198 & ST314), followed by Agona (*n* = 21/87; ST13 & ST2332), and *Salmonella*Anatum (*S*. Anatum) (*n* = 15/87; ST64). Other serotypes identified were *S. typhimurium* (*n* = 7/87; ST19 & ST36), *Salmonella* Braenderup (*S.*Braenderup) (*n* = 6/87; ST22), *S.* Weltevreden (*n* = 4/87, ST365), *S.* Newport (*n* = 3/87; ST31), *Salmonella* Indiana (*S. Indiana*) (n = 1/87; ST2040), *S. infantis* (n = 1/87; ST32), *Salmonella* Reading (*S.*Reading) (n = 1/87; ST93), *Salmonella* Rissen (*S.*Rissen) (n = 1/87; ST469), *S.* Saintpaul (n = 1/87; ST49 & ST27), and *Salmonella*Stanley(*S.*Stanley) (n = 1/87; ST2458). The isolates from pigs were more diverse and belonged to 11 different serovars. We found that 14 out of 25 human cases belonged to STs, also found in food animals. Notably, several serovars (6/16)had more than one ST, and six isolates had a unique combination of alleles not present in the database, representing new STs (Table 3).

**Fig. 2:**
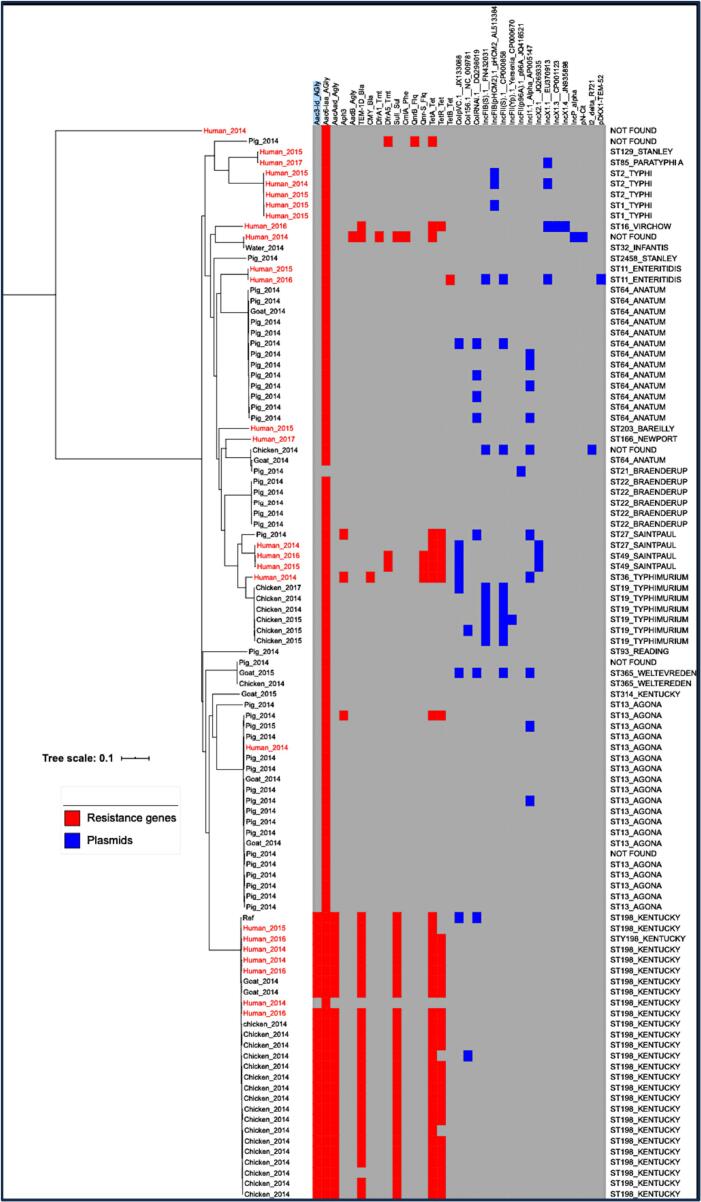
The SNP phylogenetic tree of NTS isolates with heatmap shows AMR genes (red) and plasmid replicons (blue). (For interpretation of the references to colour in this figure legend, the reader is referred to the web version of this article.)

On the resulting phylogenetic tree comprised of all sequences (Fig. 2), most serovars clustered in individual monophyletic clades; isolates did not cluster according to source or geographic location. We found that serotype and sequence types were the prime factors responsible for clustering. We additionally found close clustering of human and animal isolates across all serovars wherever isolates from both humans and animals were present. Small genetic variations (SNPs) were observed within serovars, and in certain cases, antimicrobial resistance genes varied by serovar, like in *S.* Kentucky (Fig. 2).

### 3.3 Antimicrobial susceptibility

Through MIC testing, we observed that resistance to fluoroquinolones [ciprofloxacin (28.6 %; *n* = 32/112) levofloxacin (26.8 %; *n* = 30/112)], aminoglycosides [gentamicin (24.1 %; *n* = 27/112), and tobramycin (1/112; 0.9 %)] were common; the MIC_90_ for ciprofloxacin was 12 μg/ml (Table 4). Resistance against cephalosporins [ceftazidime (1.8 %; n = 2/112), colistin (1/112; *n* = 0.9 %)], sulphonamides [trimethoprim (0.9 %; *n* = 1/112)] and azithromycin (0.9 %; n = 1/112) was less common and no carbapenem resistance was observed. Antimicrobial susceptibility was variable between serotypes. *S*. Kentucky was commonly resistant to multiple antimicrobials, with a high proportion (96.7 %; *n* = 29/30) showing resistance to fluoroquinolones, gentamicin (96.7; n = 29/30), and ticarcillin-clavulanic acid (40 %; *n* = 12/30).

**Table 4:** AMR profile of NTS.

Antibiotics	Human (%) (*n* = 25)	Animal (%) (n = 87)	Total (%) (*n* = 112)	*P*-value	MIC_50_ (μg/ml)	MIC_90_ (μg/ml)
Ciprofloxacin	10 (40 %)	22 (25.3 %)	32 (28.6 %)	0.1193	0.094	12
Levofloxacin	10 (40 %)	20 (23 %)	30 (26.8 %)	0.0779	0.25	8
Meropenem	0	0	0	0	0.25	0.25
Imipenem	0	0	0	0	1	1
Ceftazidime	2 (8 %)	0	2 (1.8 %)	**0.0482**	1	1
Cefepime	0	0	0	0	1	1
Amikacin	0	0	0	0	2	2
Gentamicin	7 (28 %)	20 (23 %)	27 (24.1 %)	0.3918	1	16
Tobramycin	1 (4 %)	0	1 (0.9 %)	0.2232	1	1
Trimethoprim/ Sulfamethoxazole	1 (4 %)	0	1 (0.9 %)	0.2232	20	20
Azithromycin	1 (4 %)	0	1 (0.9 %)	0.2232	4	8
Colistin	1 (4 %)	0	1 (0.9 %)	0.2232	0.5	0.5

P-value <0.05 was considered significant.

**Table 5:** Collection of animal samples from various regions.

**States**	**Locations**	**Approached**		**Sampled**			**No of samples collected**		
		**Farms**	**Shops**	**Chicken** **Farms/ shops**	**Pig** **Farm/ shops**	**Goat** **Farm/ shops**	**Chicken** **Stool**^**e**^**/ meat**	**Pig** **Stool**^c^**/ meat**	**Goat** **Stool**^c^**/ meat**
Haryana	Barwala	5	8	2/6	0⁎	2/2	9/6	0/0	2/2
Punjab	Patiala	9	12	2/9	2/4	1/5	12/9	7/4	2/5
	Ropar	4	22	4/15	3/7	3/15	114/15	10/7	6/18
	Mohali / Balongi	6	18	4/7	2/3	6/3	56/7	7/3	12/3
	Sangrur	3	5	2/4	1/1	0/2	10/4	3/1	0/2
	Samana	5	3	2/3	0/0	1/3	13/3	0/0	2/3
	Anandpur sahib	4	8	1/2	0/0	2/2	30/2	0/0	2/2
	Kurali	9	18	3/11	2/1	5/4	58/11	5/1	9/4
Himachal Pradesh	Nahan	7	3	4/4	0/0⁎	2/4	27/4	0/0	9/6
	Kangra	4	3	2/3	0/0⁎	2/4	7/3	0/0	5/4
	Hamirpur	5	2	1/3	0/0⁎	3/6	4/3	0/0	3/7
Chandigarh	Slaughter house			0^a^	Sampled once in a week for 30 weeks from March 2014 to October 2014		0/0	25/135	38/135
	Sector 21 market b	0	10	0/9	0^b^	0^b^	0/10	0/0	0/0
Total		61	112	27/75	8/16	27/50	340/77	57/151	90/191

⁎ No pig farms were located in this region.

a Chicken is not slaughtered at the Slaughter house, Chandigarh.

b Sector 21 market is a poultry and fish market, meat from other animals are not available here.

c Freshly passed stool samples were collected at all places except for slaughter house where we had access to intestinal contents.

Within the human isolates, 10/25 (40 %) were resistant to fluoroquinolones, 7/25 (28 %) to gentamicin, 2/25 (8 %) to ceftazidime, and 1/25 (4 %) to azithromycin, colistin, tobramycin, and trimethoprim/sulfamethoxazole. Among the animal species tested, the proportion of organisms exhibiting resistance was highest in poultry, as 75 % (*n* = 21/28) of poultry-derived organisms were resistant to levofloxacin, 60.7 % (*n* = 17/28) to ciprofloxacin, and 60 % (n = 17/28) to gentamicin. Among pig isolates, resistance was noted only to fluoroquinolones, as 4 % (n = 2/50) of organisms were resistant to ciprofloxacin, and 14 % (*n* = 7/50) were resistant to levofloxacin. Isolates from goats and sheep exhibited 22.2 % (n = 2/9) prevalence of resistance to ciprofloxacin, levofloxacin, and gentamicin. Overall, the prevalence of resistance was higher in human-derived isolates than in animal-derived isolates. Frequencies of resistance to azithromycin (*P* < 0.05), ceftazidime (*P* < 0.001), cefoperazone (P < 0.001), cefotaxime (*P* < 0.01), tetracycline (P < 0.05), aztreonam (P < 0.01) and piperacillin-tazobactam(P *<* 0.01) were found to be significantly higher among human isolates.

### 3.4 Antimicrobial resistance genes

Through analysis of whole genome sequence data, we identified 16 AMR genes in total (Fig. 2); most of which are associated with resistance to aminoglycosides, beta-lactamases, sulphonamides, phenicols, fluoroquinolones, and tetracyclines. Genes associated with resistance to aminoglycosides and tetracyclines were the most common; aac (6”-Iaa) was present in 96.6 % (*n* = 113/117) of isolates, while genes *aac*(3”-Id) and *aac-aad* were present in 24.7 % (29/117) of isolates. The latter two genes were always present together, suggesting they may be on a single genomic island. The phosphotransferase modifying gene *aph*(3”-Ia) was present in 2.6 % (2/117) of isolates. The *tet*A and tetR genes were likely responsible for tetracycline resistance and were identified in 35 % (41/117) of isolates. Regarding Beta-lactam resistance, *bla*-TEM −1 was present in 26.5 % (31/117), and *bla*-CMY was present only in two human isolates (2/117; 1.7 %). The *bla*-TEM-1 gene was present mainly in human and poultry isolates from *S*. Kentucky serovar. We found two variants of *dfr*A (*dfr*A-1 and *dfr*A-5) in 2.6 % (3/117) and a single *sul*I gene in 24.8 % (29/117) for sulphonamide resistance. Resistance to fluoroquinolones was found to be attributable to three mutations (S83F_D87G_S80I, S83F_D87N_S80I, and S83F_D87Y_S80I) in DNA *gyr*A and *par*C regions. Along with these mutations, plasmid-mediated quinolone resistance (PMQR) genes *qnr*B (1/117; 0.8 %) and *qnr*S(8/117; 6.8 %) were also present. There was a single colistin-resistant isolate. No MCR genes were detected; however, a mutation in the two-component system *Pho*P-*Pho*Q regulated protein was noted at position no three from Lysine to Asparagine (*K*—N).

### 3.5 Plasmid replicons

Seventeen different plasmid replicons were identified, mainly belonging to incompatibility group IncF type (5/17), IncX (4/17), Col types (3/17), IncP (1/17), IncI (1/17) and others (3/17). IncI alpha was the most prominent plasmid (37.5 %, *n* = 9) (Table S2, Fig. 2). Not all isolates of a particular serovar carried the same plasmid except *S. typhimurium*, where ST19 serovar specific carriage of IncFI-B and IncFII plasmid replicons was observed (Table S2, Figure2). Most isolates (85/117; 72.6 %) contained no plasmid replicons.

## 4 Discussion

Though reservoirs of NTS are food animals and those causing diarrhea in humans have been studied independently before, contemporaneous data from Northern India comparing clinical isolates of NTS with isolates from food animals collected are scarce [27,28]. Unlike Europe and the USA, where Salmonellosis is the second most frequently reported zoonotic disease with >65,000 and 1.35 to 1.4 million cases, respectively, the exact burden is unclear in India due to inadequate data [29,30]. In our study, the prevalence of NTS in humans was 1.28 %, comparable to the 1 % described by NICED in Kolkata [31]. The NTS isolates from humans were distributed across all age groups and in both sexes; NTS infections are generally mild, but the disease can be life-threatening in the young, the elderly, and the immunocompromised. Our study reported no mild infections; all NTS caused moderate to severe diarrhea. More than half of the cases (16/25; 64 %) presented with dehydration, and 40 % (10/25) patients needed hospital admission. Of all the serovars described here, *S.* Kentucky ST198 was the most common in humans, followed by *S. typhimurium*, *S.* Saintpaul, and *S. enteritidis*. Additionally, we report that *S.* Agona*, S.* Bareilly*, S.* Kentucky*, S.* Saintpaul*, and S.* Virchoware are associated with human disease for the first time in our geographic region.

In India, poultry is the most widely studied zoonotic reservoir of NTS, with information from other animals lacking [32,33]. This study sampled poultry and sheep, goats, and pigs. Although the prevalence of poultry as a reservoir for NTS has been well established, it was only the source of 32.6 % of animal-derived isolates in our study. It found a higher prevalence (56.5 %) and a higher serovar diversity (*n* = 11) in samples originating from pigs. Pigs commonly feed on sewage and can become colonized with NTS at any stage of life via horizontal or vertical transmission. Human NTS infections associated with pork consumption have increased globally in the last decade [34]. A small number of isolates from goats were obtained, and they had no serovar specificity. In poultry, only four serovars (*S.* Kentucky, *S. typhimurium, S.* Weltevreden, and *S. infantis*) were identified, and they displayed a restricted farm pattern. From other regions of India, a varied prevalence of NTS has been reported: 6 % (cattle & pigs) in Nagpur [35], 5 % (beef, mutton, pork, chicken) in Karnataka [36], 6.31 % (poultry) in Rajasthan [37] and 6.88 % (poultry) in Kashmir [38]. A higher level of contamination of meat samples in our study (8.4 %) potentially suggests a close association of NTS serovars with the food chain and high transmission risk in north India.

Some NTS serovars, such as *S.* Anatum, *S.* Bareilly, *S.* Braenderup, *S. enteritidis*, *S. Indiana*, *S. infantis*, *S.* Reading, *S.* Rissen, *S.* Stanley, and *S.* Weltevreden, belonged to a single ST type. In contrast, some human infections were caused by multiple STs, such as *S.* Virchow and *S.* Saintpaul. We found many instances where the animal isolates clustered closely with human isolates, suggesting a zoonotic link. One example was *S.* Kentucky, where the human isolates clustered with the animal isolates and exhibited an identical composition of AMR genes, highly suggestive of zoonotic transfer. We concluded that Cip^R^ *S*. Kentucky is endemic in humans in India and is likely associated with animal reservoirs, including chickens and goats [17]. In our study, all human *S.*Typhimurium belonged to ST36, not the more globally common ST19 [39]. ST19 is closely related to ST313, a clade in sub-Saharan Africa that causes invasive disease and emerged in association with the HIV pandemic [[40], [41], [42]]. Moreover, all ST19 isolates carried IncFIB(S).1__FN432031, closely related to the pSLT-BT virulence plasmid previously described in the ST313 epidemic in Sub-Saharan Africa [42]. In our collection, *S. typhimurium* ST19 organisms harboring this plasmid originated from poultry isolates, highlighting poultry as a reservoir for this variant.

*S*. Agona and *S.* Anatum were our collection's second and third most common serovars; most were isolated from pigs. These serovars are becoming increasingly associated with human infections in Europe and the USA [43]. There were two different STs of *S.* Newport represented in our collection, ST166 from humans and ST31 from animals, which mirrors what has been observed in published literature [44]. *S. infantis, S.* Virchow, and *S*. Braenderup are uncommonly reported. *S.*Braenderup is usually associated with poultry and has caused an outbreak in the USA [45]. *S. infantis* belongs to the highly conserved ST32 and is associated with clonal dissemination from food sources and humans [46]. More detailed studies are required from our geographical area to ascertain the host/ reservoir distribution of the uncommonly isolated NTS serovars.

AMR is increasing in NTS; 28 % of NTS here were MDR. Overall, the prevalence of AMR was greater in isolates from humans, with resistance to several key antimicrobials significantly associated with human isolates. Animal isolates only resisted ciprofloxacin, levofloxacin, tetracycline, and gentamicin. We hypothesize that these differences reflect different antimicrobial usage and exposure in humans and animals [47]. The most significant prevalence of resistance was observed against fluoroquinolones, most notably in isolates from poultry. This observation is especially concerning, as the Indian poultry sector is vast, with an annual growth of 8–10 % [48]. Poultry farms in the sampled geographic area are mainly mechanized farms with a capacity of 10,000 to 15,000 birds per farm. Here, the poultry is maintained in closed cages, which can facilitate the spread of organisms between birds. The emergence of fluoroquinolone resistance is critical, as it is classified as a “highest priority-critically important”antimicrobial by the WHO and is the most prescribed antimicrobial in India [49]. The unrestricted use of antimicrobials in humans and animals is likely the prime reason for developing and maintaining drug-resistant bacteria [47,50].

Resistance was variable between serovars; *S.* Kentucky was the most resistant, followed by *S. typhimurium*, *S.* Saintpaul, and *S.* Agona. All *S*. Kentucky ST198 isolates were resistant to ciprofloxacin, with resistance being associated with three mutations (S83F_D87Y_S80I) in *gyr*A and *par*C [17]. In addition to chromosomal mutations, the plasmid-mediated quinolone resistance genes (PMQR) *qnr*B and *qnr*S were also identified. These genes were mainly detected in human and pig isolates, indicating that both resistance mechanisms are prevalent in our region. As observed previously, *bla-*TEM was the most frequently occurring beta-lactamase, present mainly in inhuman isolates; this gene was only present in *S.* Kentucky from animals. We additionally identified the *bla*-CMY beta-lactamase gene in two human isolates. The *bla*-CMY was not found in animal isolates, but this gene has been reported previously in pigs from India's northeastern parts (Mizoram, Meghalaya, and Assam) [51].

## 5 Conclusion

This study is the first genomic analysis from India at this scale to study the zoonotic reservoirs of NTS organisms. We report a rich diversity of *Salmonella* serovars that infect humans in this geographic area, with the majority having reservoirs in food animals. Among the different food-producing animals, pigs appeared to be a key source of human infections. *S. typhimurium* ST19 harboring the plasmid IncFIB (S).1__FN432031 originated from poultry isolates, highlighting poultry as a reservoir for this variant. The most significant prevalence of resistance was observed against fluoroquinolones, most notably in isolates from poultry. A high level of contamination of meat samples in our study (8.4 %) potentially suggests a close association of NTS serovars with the food chain and high transmission risk in north India.

## Funding information

A visiting fellowship granted by OUCRU supported the work, Ho Chi Minh City, Vietnam, to J. M. This research was supported by an 10.13039/501100001411Indian Council of Medical Research (ICMR) grant to the PGIMER (Grant Number: 5/8–1(37)2012–13 ECDII) and WHO-AGISAR India (Grant reference number 2013/315402–0 and Reg File No. F9-TSA-0001).

## Author contributions

JM: Conceptualization, Data curation, Investigation, Methodology, Manuscript writing, review, and editing.

DPT: Resources, Software, Investigation and Formal Analysis,

HK: Methodology, Formal Analysis, Manuscript writing, review, and editing.

TNTN: Formal analysis and software

TN: Software, Methodology, and Formal analysis.

CRE: Review and Editing.

THTN: Software, Methodology, and Formal analysis.

RV: Resources.

BM: Supervision.

ST: Supervision and Funding.

SB: Supervision, Funding, and Manuscript review and editing.

NT: Conceptualization, Investigation, Supervision, Funding and Manuscript writing, review and editing.

## Ethical statement

This study was approved by the Institutional Ethics Committee, PGIMER, Chandigarh, India, reference number NK/4458/PhD, and the PGIMER Collaborative Committee, reference number 79/227-Edu-18/4997. All human samples included in this study were collected after obtaining informed consent from the patient or their guardian.

## Consent to publish

Written permission was obtained from all participants/guardians during sample collection as part of informed consent.

## Data summary

Raw genomic reads of non-typhoidal *Salmonella* isolates have been deposited in GenBank under the accession number PRJEB47524.

## CRediT authorship contribution statement

**Jaspreet Mahindroo:** Conceptualization, Data curation, Formal analysis, Investigation, Methodology, Writing – original draft, Writing – review & editing. **Duy Pham Thanh:** Formal analysis, Investigation, Resources, Software. **Harpreet Kaur:** Formal analysis, Methodology, Writing – original draft, Writing – review & editing. **To Nguyen Thi Nguyen:** Formal analysis Software. **Trang Hoang Thu Nguyen:** Formal analysis, Methodology, Software. **Megan E. Carey:** Review and Editing. **Ritu Verma:** Resources. **Balvinder Mohan:** Supervision. **Siddhartha Thakur:** Funding acquisition, Supervision. **Stephen Baker:** Funding acquisition, Supervision, Writing – review & editing. **Neelam Taneja:** Conceptualization, Investigation, Project administration, Resources, Supervision, Writing – original draft, Writing – review & editing.

## Declaration of competing interest

The authors declare that they have no known competing financial interests or personal relationships that could have appeared to influence the work reported in this paper.

## Data Availability

No data was used for the research described in the article.

## Appendix A Supplementary data

Supplementary material Image 1
