# Supplementary material for: The genomic diversity and antimicrobial resistance of Non-typhoidal Salmonella in humans and food animals in Northern India
Source: One Health. 2024 Sep 12;19:100892. doi: 10.1016/j.onehlt.2024.100892 (PMC11439553; doi:10.1016/j.onehlt.2024.100892)
Supplement: Supplementary file 1 — Supplementary material [file mmc1.pdf]

Table S1. Clinical details of patients samples included in this study

S. no	Year of isolation	Serovar	Location	Age group	Diagnosis	Fever	Pain abdomen	Vomiting	Dehydration	Hospitalization	Duration of diarrhoea at time of presentation	Vesikari score	Severity
1	2017	Kentucky	Manimajra, Chandigarh	<5 years child	Acute gastroenteritis	+	+	-	+	-	7	9	Moderate
2	2016	Kentucky	Chandigarh	<5 years child	Acute gastroenteritis	+	+	+	+	+	2	14	Severe
3	2014	Typhi	Chandigarh	>5 years child	Pyrexia of unknown origin with diarrhoea	+	+	+	+	+	5	15	Severe
4	2015	Typhimurium	Kishangarh, Haryana	<5 years child	Acute gastroenteritis	+	+	-	+	-	3	7	Moderate
5	2015	ND	Panchkula, Haryana	>5 years child	Acute foodborne gastroenteritis	+	-	+	-	-	2	8	Moderate
6	2014	Agona	Chandigarh	Adult	Acute foodborne gastroenteritis	+	+	+	+	+	7	13	Severe
7	2017	Newport	Nawashaher, Punjab	Adult	Acute foodborne gastroenteritis with neutropenia	+	+	+	+	+	1	15	Severe
8	2015	Typhimurium	Manimajra, Chandigarh	<5 years child	Undifferentiated fever with diarrhoea	+	+	+	+	+	5	14	Severe
9	2015	Paratyphi A	Moli Jagran, Chandigarh	<5 years child	Acute gastroenteritis	+	-	-	+	-	10	8	Moderate

10	2014	Infantis	Ambala, Haryana	Adult	Acute foodborne gastroenteritis	+	+	+	+	+	2	10	Moderate
11	2015	Typhi	Chandigarh	Adult	Fever with Acute gastroenteritis	+	+	+	-	-	1	8	Moderate
12	2015	Kentucky	Nawan Shahar,Punjab	Adult	Acute gastroenteritis with splenomegaly	+	+	+	-	-	2	8	Moderate
13	2015	Typhi	Manimajra, Chandigarh	Adult	Fever with acute gastroenteritis	+	+	+	-	+	3	12	Severe
14	2016	Saintpaul	Manimajra, Chandigarh	Adult	Acute foodborne gastroenteritis	-	+	+	+	-	2	8	Moderate
15	2016	Enteritidis	Rishikesh, Uttarakhand	<5 years child	Acute gastroenteritis	-	-	+	+	-	3	9	Moderate
16	2017	Paratyphi A	Chandigarh	Adult	Dysentery	+	+	_	_	-	7	11	Severe
17	2014	Kentucky	Amritsar, Punjab	Adult	Acute myeloid leukaemia with acute gastroenteritis	-	+	+	+	+	2	18	Severe
18	2015	Bareilly	Panchkula, Haryana	>5 years child	Acute foodborne gastroenteritis	-	+	+	-	-	4	8	moderate
19	2017	Kentucky	Chandigarh	Adult	Foodborne gastroenteritis	+	+	+	-	-	3	9	Moderate
20	2014	Virchow	Karnal, Haryana	Adult	Post transplant dysentery	+	+	+	-	+	2	14	Severe
21	2015	Kentucky	Mohali, Punjab	Adult	Acute gastroenteritis with chronic kidney disease	+	+	+	+	+	3	14	severe

22	2014	Typhimurium	Manimajra, Chandigarh	<5 years child	Acute gastroenteritis	-	+	+	+	+	5	11	Severe
23	2015	ND Anatum by serotyping	Noorpur, Haryana	Adult	Dysentery	+	+	+	+	+	7	14	Severe
24	2015	Saintpaul	Manimajra, Chandigarh	<5 years child	Dysentery	+	+	+	+	-	1	10	Moderate
25	2015	Kentucky	Chandigarh	Adult	Acute foodborne gastroenteritis	+	+	+	+	+	2	13	Severe
26	2016	Saintpaul	Kalka, Haryana	Adult	Acute foodborne gastroenteritis	+	+	-	+	-	4	9	Moderate
27	2015	Typhi	Chandigarh	Adult	Fever with diarrhoea	+	+	-	+	+	4	12	Severe
28	2014	ND	Chandigarh	Adult	Foodborne gastroenteritis	+	+	+	-	-	3	10	Moderate
29	2016	Virchow	Shimla, Himachal Pradesh	Adult	Acute foodborne gastroenteritis	+	+	+	-	-	2	9	Moderate
30	2014	Kentucky	Nayagaon, Punjab	Adult	Acute foodborne gastroenteritis	+	+	+	-	-	3	10	Moderate
31	2015	Entertidis	Zirakpur, Punjab	Adult	Foodborne acute gastroenteritis	+	+	+	+	+	2	14	Severe

ND= Not determinted by WGS

Supplementary Table S2: Plasmid replicon types and incompatibility groups in NTS

Incompatibility group	Type	n
IncF	IncFIB(S).1	6
	IncFIB(pHCM2).1	2
	IncFII(S).1	6
	IncFII(Yp).1	1
	IncFII(p96A).1	1
IncX	IncX2.1	3
	IncX1.1	1
	IncX1.3	1
	IncX1.4	1
IncP	IncP_alpha	1
IncI	IncI1.1_Alpha	9
Colicins	ColpVC.1	6
	Col156.1	1
	ColRNAI.1	4
Others	pN-Cit	1
	I2_delta	1
	pDKX1-TEM-52	1
Total	17	

Supplementary Table S3: Sequence parameters used during SNP tree generation

Isolate	Cover%_NZ_CP026327	Depth_NZ_CP026327	Mapped%_NZ_CP026327	Mapped%_Total	Total_Reads	SNPs	Hets_Removed	Indels	Ingroup/Fail
782-14	14.51714	8.829324	7.172666734	7.172666734	751436	318	13	0	f
P11	90.75372	41.14509	91.64515637	91.64515637	1427998	42677	253	365	i
P15	91.84979	8.494584	95.56866211	95.56866211	488814	36117	323	247	f
C164	91.10935	18.75978	88.78949654	88.78949654	1027242	34005	287	261	i
C73	98.40633	16.32032	98.14985345	98.14985345	510392	172	2	8	i
P28	87.23214	16.14439	92.27597179	92.27597179	751758	35277	422	238	i
57-14	90.29811	7.608945	93.2037507	93.2037507	394806	38687	379	226	f
S-207	98.52406	13.68337	97.92869757	97.92869757	738376	187	2	9	i
M706	98.42721	16.05987	98.96610961	98.96610961	505276	176	2	6	i
2447-15	89.25027	17.28663	91.65236814	91.65236814	911516	46407	512	365	i
PM118A	91.10904	14.56767	93.17105767	93.17105767	805132	33189	486	202	i
840-14	83.31558	14.2888	81.04041153	81.04041153	790418	117553	2034	447	o
GM121	92.24623	33.63864	96.43088871	96.43088871	1189820	41921	145	350	i
M747	91.61668	22.53296	91.1025156	91.1025156	707346	38847	128	269	i
PM33	91.78863	25.10817	91.45107013	91.45107013	744678	35364	402	279	i
PM32	91.01984	14.95053	90.58162961	90.58162961	804120	41670	635	317	i
P7	90.12433	12.97797	91.41317644	91.41317644	712848	45935	313	331	i
PM116	90.97974	12.93912	93.51692647	93.51692647	706054	46939	348	339	i
PM75	90.18918	7.643145	91.650593	91.650593	397130	38618	377	254	f
P34	91.75071	66.96281	91.94748788	91.94748788	2343840	43455	749	381	i
P13	91.78059	14.54977	89.59183284	89.59183284	838284	41961	574	333	i

HS23	90.57873	15.26338	90.91226637	90.91226637	827368	45067	1035	321	i
PM117	90.95617	13.48771	93.51988987	93.51988987	842640	46525	293	331	i
C157	98.38288	7.649235	99.03402771	99.03402771	395560	110	0	8	f
C143	98.49522	14.42387	99.11893818	99.11893818	774066	202	7	9	i
C144	98.48696	18.609	99.09542729	99.09542729	985106	208	2	8	i
389-17	14.30523	1.424881	50.80701754	50.80701754	632700	0	0	0	f
PM30	91.63405	12.68874	88.13831591	88.13831591	707800	41080	1035	304	i
G18	96.82152	20.18266	97.71345293	97.71345293	620980	183	10	6	i
P3	88.93545	13.71014	93.23962063	93.23962063	708762	46450	781	351	i
PM34	90.06548	17.57735	89.82096027	89.82096027	952526	46176	605	334	i
PM130	90.99274	12.76989	91.23493564	91.23493564	423682	32831	3111	255	i
522-17	90.18249	30.13179	95.16008643	95.16008643	841358	39349	120	297	i
C181	92.60386	102.2975	86.0737	86.0737	4021384	31503	36665	288	i
P16	90.78429	8.28792	89.4549058	89.4549058	447042	35625	388	245	f
G24	91.20365	34.19333	92.95501631	92.95501631	975020	37228	150	304	i
C134	98.46604	11.2012	99.14243662	99.14243662	693010	177	2	9	i
M278	91.39272	10.44613	90.57414739	90.57414739	563270	44860	341	304	i
G25A	89.88124	17.52268	91.43139886	91.43139886	473928	39441	432	265	i
P47	90.78612	8.846728	90.88955476	90.88955476	477902	36950	866	240	f
C147	98.52234	42.25007	99.10764659	99.10764659	1460520	248	19	7	i
M277	90.55487	17.53273	88.1197626	88.1197626	518626	39437	351	243	i
830-15	87.23148	18.6976	87.46485838	87.46485838	1027272	55237	356	541	i
PM150	89.88467	18.09222	94.00210779	94.00210779	601578	33772	312	224	i
C139	98.16757	14.75621	98.95736741	98.95736741	446466	172	2	4	i
688-15	87.45549	46.82195	88.82191589	88.82191589	1626692	56436	213	592	i
M270	90.37082	8.377021	86.5117308	86.5117308	465612	40845	401	283	f
PM115	90.86744	8.609837	93.40051093	93.40051093	443898	41821	466	272	f
1496-14	91.3106	13.05655	90.90218773	90.90218773	750642	44559	743	343	i
P25	90.37297	16.2638	90.61060223	90.61060223	876872	46123	858	328	i

M363	89.44564	17.66491	94.93128223	94.93128223	479352	38645	282	249	i
PM118B	90.9868	14.93497	93.65769771	93.65769771	835028	47637	306	356	i
C89	90.93742	13.57419	89.03733317	89.03733317	743414	46949	690	365	i
C138	90.00205	12.509	89.11168259	89.11168259	680500	42125	340	319	i
C78	98.50134	14.36255	98.28447312	98.28447312	768860	203	2	9	i
PM119	91.63001	14.89502	90.27390029	90.27390029	814530	41334	1034	316	i
C161	98.48061	11.58905	98.85743685	98.85743685	618084	123	4	5	i
P42	91.18914	79.94231	91.79279917	91.79279917	3044814	43354	219	373	i
C186	90.8664	10.40614	88.78429616	88.78429616	593944	45371	364	334	i
P8	89.8327	14.88891	92.10735881	92.10735881	796134	46140	294	311	i
C137	98.50679	17.0632	99.08028823	99.08028823	913982	213	3	9	i
P17merge	89.8691	18.23817	89.63316118	89.63316118	956878	49922	565	384	i
PM122	90.88246	15.23541	95.51710013	95.51710013	424792	37673	377	248	i
C133	98.22675	17.09589	99.16766487	99.16766487	422426	227	1	9	i
M361	87.78383	20.95786	95.45815029	95.45815029	547244	43851	255	338	i
S-783	91.59499	18.20322	90.48691081	90.48691081	986998	42105	752	329	i
C162	98.49767	16.22575	98.85704845	98.85704845	865566	195	2	7	i
76814	98.43861	16.99955	98.06781854	98.06781854	476094	166	2	7	i
PM143	90.09335	24.19117	93.63839878	93.63839878	753128	37671	195	266	i
C136	90.09574	8.457764	89.02347476	89.02347476	452912	36560	524	258	f
H243	98.21881	16.30011	97.45163065	97.45163065	879896	149	0	6	i
PM129	90.89285	16.54687	91.58537048	91.58537048	1000662	41867	244	334	i
PM146	90.10562	54.49616	92.28817093	92.28817093	1961558	47483	169	362	i
GM124	90.75006	86.82271	87.50924816	87.50924816	3581794	44516	118	386	i
P46merge	90.85073	19.46875	93.17910469	93.17910469	990002	42349	415	347	i
P32	91.68463	55.25626	92.14321378	92.14321378	1998858	43336	733	368	i
PM92	90.59401	12.58866	86.51338597	86.51338597	715376	42103	932	315	i
UR34merge	89.77264	14.96303	92.29408906	92.29408906	753214	46164	535	361	i
C75	98.51265	17.52539	98.52967239	98.52967239	1263936	185	0	9	i

C148	98.52214	20.19263	98.58803895	98.58803895	1089336	177	2	6	i
PM132	90.86797	11.75192	91.47790853	91.47790853	664872	40781	343	302	i
C141	98.34609	18.33928	99.09642527	99.09642527	576156	205	2	8	i
1511-15	87.44298	14.98705	86.83525292	86.83525292	814934	53885	1168	518	i
P38	90.7045	15.68415	91.96214931	91.96214931	829998	46669	523	344	i
C155	98.50498	19.19555	98.69672328	98.69672328	992882	191	3	8	i
PM120	91.3781	9.350859	89.41809228	89.41809228	570984	38090	465	287	f
1217-15	89.62793	7.041332	92.26431391	92.26431391	361222	35309	375	226	f
C145merge	98.51022	13.5	98.10469132	98.10469132	694346	188	35	6	i
G26	98.51324	12.77868	97.36337098	97.36337098	687848	192	2	7	i
P4merge	91.91868	21.09288	92.84824335	92.84824335	1093158	42854	337	345	i
Jun-17	87.45163	17.78711	92.51599166	92.51599166	912038	52625	448	446	i
G25merge	90.26091	17.66755	89.01617971	89.01617971	954158	47206	647	346	i
PM144	89.94387	20.68151	93.12967405	93.12967405	1102364	46687	335	339	i
G52B	89.61783	9.78053	85.7762451	85.7762451	546902	42642	452	282	f
1718-15	87.29544	14.96748	90.28944181	90.28944181	396280	44912	665	393	i
PM149	89.89871	14.97479	94.12696842	94.12696842	768002	45748	598	318	i
1414-16	98.51671	19.35424	97.15612504	97.15612504	1044842	231	6	7	i
M494	91.61752	15.54957	87.88932006	87.88932006	770510	35614	495	226	i
C180	91.05135	25.69842	89.44344686	89.44344686	1387792	48660	242	408	i
P5	91.10406	15.51749	90.77501327	90.77501327	727394	31561	492	233	i
PM13	90.3907	20.02551	88.68138752	88.68138752	613008	43372	945	288	i
102-14	87.059	14.52202	88.63868484	88.63868484	468018	42133	563	335	i
G27merge	91.05251	14.40283	91.45567696	91.45567696	756514	41891	550	321	i
PM151	89.82915	16.27619	92.13869383	92.13869383	938470	45990	322	330	i
PM31	91.71735	15.90018	87.96073419	87.96073419	898186	42133	866	321	i
1427-15	98.50649	11.78445	99.17162773	99.17162773	625202	170	3	6	i
P56	89.84153	16.83799	92.31981215	92.31981215	1085950	45645	225	329	i
W13	90.80247	12.80838	93.20804357	93.20804357	673532	44296	900	334	i

C140	98.45233	12.20984	98.93065749	98.93065749	643386	191	2	7	i
P50	91.08608	32.87122	93.48423638	93.48423638	949528	35081	176	284	i
1185-16	98.47602	10.82653	97.81319585	97.81319585	583820	191	5	6	i
PM114	89.83633	14.1222	90.45372914	90.45372914	757280	45747	536	311	i
C91	90.8711	13.12157	88.92379736	88.92379736	804644	46947	306	381	i
CM11	91.29889	17.42717	90.46549017	90.46549017	528690	37832	332	264	i
